# Development of a self-administered questionnaire to identify levers and barriers to adherence to medication regimens for chronic disease: The QUILAM project

**DOI:** 10.1371/journal.pone.0323542

**Published:** 2025-10-23

**Authors:** Audrey Lehmann, Carine Meslot, Matthieu Roustit, Jean-Didier Bardet, Aurélie Gauchet, Benoît Allenet

**Affiliations:** 1 University. Grenoble Alpes, TIMC, CNRS UMR5525, VetAgro Sup, Grenoble INP, Grenoble, France; 2 University Grenoble Alpes, Pôle pharmacie, Centre Hospitalo-Universitaire Grenoble Alpes, Grenoble, France; 3 University Savoie Mont Blanc and University Grenoble Alpes, LIP/PC2S, Grenoble, France; 4 University Grenoble Alpes, INSERM U1300, VetAgro Sup, Grenoble INP, Grenoble, France; 5 University Grenoble Alpes, INSERM CIC1406, Centre Hospitalo-Universitaire Grenoble Alpes, Grenoble, France; National Tuberculosis Institute, GUINEA

## Abstract

**Objective:**

We aimed to develop a generic questionnaire for use in routine clinical practice to identify barriers and levers to medication adherence, particularly for patients with chronic disease, in order to implement targeted interventions.

**Materials and methods:**

To generate items we conducted a narrative literature review and qualitatively analyzed semi-structured interviews of patients with chronic diseases (type 2 diabetes, chronic obstructive pulmonary disease and heart failure). Items were classified according to the five dimensions that influence medication adherence proposed by the WHO. Qualitative reduction of the number of items was conducted by a panel of experts. This long questionnaire was tested on ambulatory patients and analysis of responses enabled quantitative item reduction. Validation of the short questionnaire and stability over time was tested on an independent cohort of patients, and again 15 days later.

**Results:**

The 194 items extracted from the literature review (validated in several pathologies) and/or from analysis of 45 interviews, spanned all five dimensions of medication adherence according to the WHO. The expert panel reduced the number of items to 62. The analysis of responses from 112 patients using factorial component analysis reduced the final questionnaire to 14 questions each scored on a 7-point Likert-type scale. A validated visual analogue scale (0–100) was added to evaluate medication adherence in general. The final version was well accepted by patients and took 5–10 minutes to complete, with little missing data. In an independent community pharmacy population (n = 55) concordance of the results between day 0 and day 15 was acceptable.

**Conclusion:**

This generic questionnaire (initially developed in French) for the identification of barriers and levers to medication adherence in patients with chronic disease could help care teams to structure and propose help-with-adherence visits to their patients and personalize interventions to improve patient outcomes.

## Introduction

Worldwide, chronic diseases are responsible for more than 70% of all deaths [1]. Clinical outcomes of patients with chronic disease depend largely on medication adherence [2–5], yet, in Western countries adherence to long-term therapy for chronic conditions averages only 50% [6,7], although these estimates vary across conditions. Medication nonadherence is considered as a silent global epidemic that leads to major economic and public health problems for healthcare systems [8–10].

Medication adherence is a dynamic behavior, variable in time and form [11]. It is defined according to three operational and quantifiable parameters: Initiation, Implementation, and Persistence [12,13]. While initiation is a discontinuous or binary process for which a yes or no answer can be given, implementation and persistence are continuous behaviors. Twenty to thirty percent of patients never initiate their prescribed treatment [14]. Implementation is inconsistent even for patients who continue to take their treatment [15], and finally half of the prescription drugs dispensed each year are not taken as prescribed [14]. Non-persistence with medicines prescribed for long-term conditions is often problematic with rates of non-persistence increasing steadily from initiation to 2 years [8,16].

Intentional non-adherence arises from the beliefs, attitudes and expectations that influence patients’ motivation to begin and continue their treatment regimen. It is subject to cognitive and emotional barriers. Unintentional non-adherence results from capacity and resource limitations that prevent patients from implementing their decision to follow treatment recommendations and involves individual constraints and circumstantial aspects. It reflects practical barriers [17]. Patients can and often do report both forms of non-adherence simultaneously or according to life events [18].

There is no gold standard for the assessment of medication adherence. Ideally, a simple, valid and reliable method for detecting prevalence and types of non-adherence is needed [17]. Such a method should be reproducible and sensitive to changes in adherence. While many methods are available to assess patient adherence, each has its own specific limitations and none is necessarily better than another [19]. Each method appears to capture different information about medication adhesion [20]. On one hand direct methods provide evidence of drug intake; on the other hand, indirect methods provide proxy data capturing patient behavior as accurately as possible. Triangulation of the different methods makes it possible to get closer to the patient’s true medication adherence behavior [20,21].

Finally, there is a plethora of tools to measure medication adherence, often built on a populational perspective, for clinical research and focused on the quantification of the phenomenon. In our opinion none of them meet the conditions required, which are: 1) allow to identify patients at risk of drug non-adherence in routine clinical practice; 2) pinpoint the major dimensions associated with this non-adherence, so that the healthcare team can adapt the patient’s care and initiate targeted intervention; 3) present acceptable measurement properties according to the COSMIN Checklist [22]; 4) suggest interventional input for each barrier identified.

As mentioned by Stewart et al, “*a key priority for future research should be to identify modifiable factors relating to nonadherence which can then be the focus of adherence support interventions at the level of the individual*” [14].

Therefore, we aimed at constructing a generic tool for use in routine clinical practice (simple, self-administered, suitable for both inpatient and outpatient practice) to monitor intentional and unintentional medication non-adherence based on the 3 processes of the ABC taxonomy (initiation, implementation and persistence) whatever the chronic disease. Unlike existing tools (most of them developed for epidemiology and with a populational perspective), our emphasis was on the individual, trying to qualify the phenomenon by identifying modifiable barriers and levers with the perspective of implementation of health interventions. Here, we present the construction of the questionnaire, its reliability and preliminary psychometric properties.

## Methods

### 1. Ethics Statement

Ethical approval for this single center study was obtained on February 25, 2013 (CECIC Rhône Alpes-Auvergne, Clermont-Ferrand, Institutional Review Board 5891). In the development phase, all eligible patients were informed about the study and gave written informed consent before being included. Likewise, for the validation test-retest phase, all community pharmacy clients were informed about the study and gave written consent to participate before being given the questionnaire to complete.

### 2. Study design

**Fig 1:**
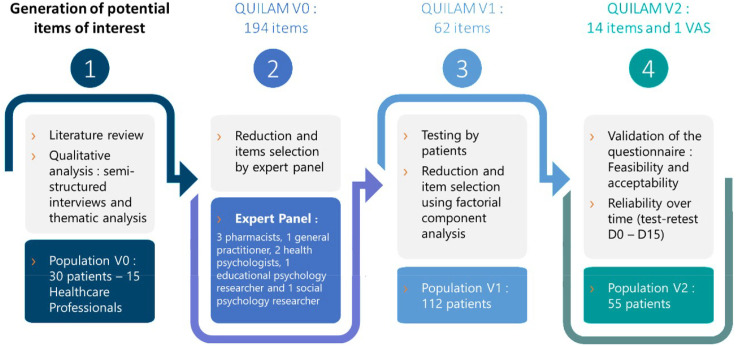
Flow chart of the construction and the validation of the QUILAM questionnaire.

The QUILAM project was divided into four steps: 1) Generation of potential items of interest (literature review and qualitative interviews); 2) Qualitative reduction by an expert panel using the Delphi method [23]; 3) Testing by patients and quantitative reduction using factorial component analysis; 4) Validation of the questionnaire and stability over time in an independent patient population (see Fig 1).

### 3. Literature review

A narrative review was conducted in PubMed and PsycInfo databases to identify self-reported scales measuring drug adherence, and validated in several chronic diseases. The following MESH terms were used to search databases: (“medication adherence” OR compliance OR adherence OR observance* OR adhesion OR concordance OR capacitance) AND (measure* OR *assess*OR self-report*). The following search criteria were checked: “Validation study” for article type, “Humans” for population, “English” and “French” for language. The criteria for inclusion in the selection were articles validating a questionnaire assessing drug adherence in patients with chronic diseases up to 2013. The questionnaire had to be validated in at least two chronic conditions. A manual search of references cited in the included articles was also conducted to complete the literature search.

### 4. Patient interviews for qualitative data collection

Semi-structured interviews were conducted with 10 patients (ambulatory or hospitalized) and 5 healthcare professionals for each pathology targeted, i.e., type 2 diabetes, chronic obstructive pulmonary disease (COPD) and heart failure. We chose to target these three major and highly prevalent emblematic chronic conditions, in order to address a broad spectrum of barriers, also recognizing that with these conditions, patients could have several co-morbidities. Interviews were conducted informally (between March 25, 2013 and December 20, 2013) using a semi-structured grid of questions asking the patients and healthcare professionals about the barriers and levers to medication adherence (see Interview Guide in supporting information S1 File). All participants gave written informed consent to be interviewed and for the interviews to be recorded and then transcribed for analysis.

A thematic analysis of the semi-structured interviews was conducted to complement the themes extracted from the literature review. This consisted of classifying the responses according to the five WHO dimensions [7], i.e., disease, drug treatment, demographic and socio-economic factors, the patient and/or their entourage, and the health care system, using a grid derived from Baudrant et al. [11]. Coding was carried out by 1 pharmacist and 1 psychologist working independently. If consensus was not reached, a third expert helped finalize the classification.

### 5. Qualitative item reduction

Firstly, a preselection of items was carried out independently by five of the project investigators (3 pharmacists, 2 psychologists) with the instruction to include the five main WHO dimensions [7], and a maximum of 2 items per sub-dimension from the grid proposed by Baudrant et al. [11]. The latter contains 5 sub-dimensions for disease, 6 for medications, 5 for demographic and socio-economic factors, 2 for the health care system and 9 about the patient and their entourage.

Secondly, the preselection was then presented to a panel of 8 experts composed of 3 pharmacists, 1 general practitioner, 2 health psychologists, 1 educational psychology researcher and 1 social psychology researcher. The experts used a Delphi method [23] to select the most relevant and informative items. Items selected by more than 3 experts were retained. Other items were retained or excluded after discussion and consensus.

As the patient population was French, the translation into French of questions that were initially in English and not available in a validated French questionnaire was done by 2 investigators working independently (1 pharmacist and 1 psychologist), with back translation. The expert committee validated the translations.

A question was added at the end of the questionnaire to assess clarity and ease of completion: “In your opinion, is this questionnaire clear and easy to fill in?” Participants could answer by ticking the corresponding box “yes, entirely”, “quite”, “moderately”, “not very” or “no, not at all”.

### 6. Testing long questionnaire (V1) by patients and quantitative item reduction

#### Patient population.

We selected ambulatory patients with frequent chronic diseases, i.e., type 2 diabetes, chronic obstructive pulmonary disease (COPD) or heart failure attending Grenoble-Alpes University Hospital between June 10, 2014 and January 28, 2015 to test the long version of the questionnaire containing all items (questions) retained by the expert panel (V1). Participants were included by clinical pharmacists and psychologists in different departments (endocrinology, pneumonology, cardiology) of the hospital. Patients participated in the study voluntarily, without any financial compensation. The responses were analyzed with the goal of reducing the number of questions.

### Quantitative item reduction

Items not meeting the acceptability criterion (frequency of complete responses defined as completion of the item by >95% of patients) were removed.

Descriptive statistics were performed for each item to test the normality of distribution, evaluate floor and ceiling effects. Items with a very inhomogeneous distribution were removed.

Likewise, a correlation matrix was constructed to eliminate items that were too highly correlated (Pearson’s correlation coefficient >0.9).

Details of the analysis are as follows: We chose an initial structure, reconstructed and KMO (Kaiser – Mayer – Olkin) indices for the descriptive part, an extraction with maximum likelihood, a factorial structure without rotation and a collapse plot, a direct Oblimin rotation and a “structure after rotation”, by classifying the variables by size and by removing the small coefficients lower than.30. The Oblimin rotation, a type of oblique rotation, was chosen, because of the presumption of correlation of the factors [24]. The items were then removed one by one according to 2 criteria: saturation on several or no dimension(s) and low saturation on one dimension (<.300). Internal consistency of the different dimensions was assessed using Cronbach’s alpha.

After quantitative data reduction a visual analogue scale (VAS) designed to assess overall medication adherence was added, Kalichman et al. [25]. This VAS (with instructions in French) had been previously tested and validated [26]. Patients were asked to place a cross on the VAS (from 0 to 100) to estimate their medication adherence, with 0 representing never taking their medication and 100 representing always taking it at the prescribed times and doses. Kalichman’s single item VAS was chosen because it correlates very strongly with objective measures of medication adherence, more so than other self-reported measures, which can overestimate adherence [25].

### 7. Validation and Reliability by test-retest

The final version of the questionnaire (V2) was tested on patients attending volunteering selected community pharmacies. Between October 14, 2019 and March 13, 2020, the study community pharmacists proposed participation in the study to ambulatory patients with a frequent chronic disease (type 2 diabetes, chronic obstructive pulmonary disease (COPD) or heart failure). Participants were asked to complete the paper questionnaire (alone without assistance) twice, on inclusion in the study and 15 days later. Some questions were posed differently such that the answers on the Likert scale was inverted, to avoid an order effect bias.

Responses to the final version of the questionnaire (V2) was assessed for reliability over time (15 days) using the intra-class correlation coefficient (ICC) with a graphical representation according to Bland-Altman’s method [27]. Statistical analyses were performed with SPSS v25 software (IBM Corp, USA).

For stage 3, a sample of 120 subjects each responding to 3 or 4 items achieves 90% power to detect the difference between the actual coefficient alpha of 0.7 and the coefficient alpha under the null hypothesis of 0.5 (since Cronbach’s alphas <0.5 are considered too low to be reliable) using a two-sided F-test with a significance level of 0.05. For the test-retest phase, we anticipated that a random sample of 60 subjects who are each measured 2 times produces a two-sided 95% confidence interval with a width of about 0.25 when the estimated intraclass correlation is 0.7, analyzed using a two-way mixed-effects ANOVA model. Sample size calculations were performed with PASS v22 (NCSS, Kaysville, Utah, USA).

### 8. Data collection on patients answering questionnaire V1 or V2

The following data were collected about the patients who participated in the quantitative reduction stage (V1) and the validation (test-retest) stage (V2): patient characteristics (age, sex/gender, level of education, marital status), disease data (disease, time since diagnosis) and drug therapy data (need for assistance to manage medications, number of drugs prescribed for the chronic disease, time since the treatment was started, concomitant drugs). The self-administered questionnaires were left for the patient to complete alone and collected after a few minutes or later. The time to complete the questionnaire was of 5–10 minutes.

Although our principal objective was not an adherence score, post-hoc analyses were carried out on the test – retest data comparing the French version of the Kalichman VAS on D0 and on D15, the total QUILAM score and the scores from the QUILAM dimensions. Two types of score calculation were performed for each patient. First, a global score was calculated using the sum of the scores for each item, but with responses to questions 1, 2, 3, 5, 6, 8, 9, 10, 11, and 14 recoded as the questions had been inversed to avoid a filling/order effect bias. The lower the total score, the greater the risk of medication nonadherence for the patient. Then, a score per dimension was calculated based on the sum of the scores for each item within each dimension.

## Results

### 1. Generation of self-reported items

#### Literature review.

**Fig 2:**
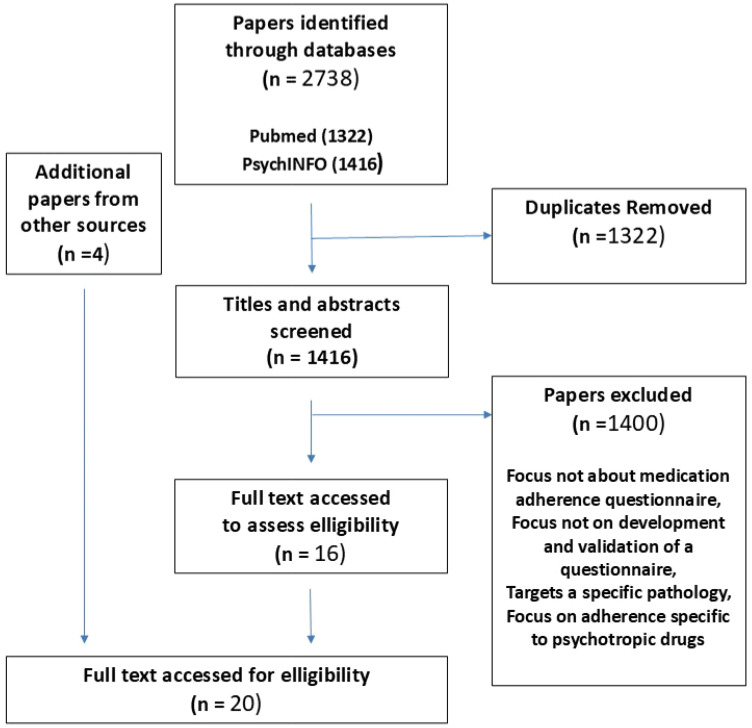
Literature search flow chart.

The literature search process is shown in Fig 2. Twenty articles were retrieved using database and manual searches giving a total of 194 items (see Table 1). The items found in the literature classified by the WHO criteria are shown in Table 2. All the themes and sub-themes not specific to a particular disease were indeed addressed in the questionnaires found in the literature.

**Table 1:** Validated questionnaires retained in literature search and number of relevant items.

Included questionnaires validated for at least 2 non-specific pathologies	Reference*	Number of items
Adherence estimator (McHorney et al., 2009)	19	3
ARMS/ Adherence to Refills and Medications Scale (Kripalani et al., 2009)	5	13
ASK20 – ASK12 (Hahn et al., 2008/ Matza et al., 2009)	9,10	20
ASRQ/ Adherence Self-Report Questionnaire (Schroeder et al., 2006)	16	7
BMQ/ Beliefs about Medicine Questionnaire (Gauchet et al., 2007; Fall et al., 2014) French version	17,18	18
Choo et al. Questionnaire, 1999	6	5
DAI-10/Drug Attitude Inventory (Hogan et al., 1983)	20	10
Girerd et al., 2009 in French	12	6
MAQ/Medication Adherence Questionnaire/Morisky 4 (Morisky et al., 1986)	1	4
MARS/Medication Adherence Rating Scale (Thompson et al., 2000)	2	10
MAR-Scale/ Medication Adherence Reasons Scale (Unni et al., 2013)	11	20
MASRI/ Medication Adherence Self-Report Inventory (Walsh et al., 2002)	14	11
MAS/ Medication Adherence Scale (Brooks et al., 1994)	4	6
MMAS/ Morisky Medication Adherence Scale)/Morisky 8 (Korb-Savoldelli, 2012) in French	3	8
MOS/ Medical Outcome Study (Kravitz, 1993)	23	5
RAMS/ Reported Adherence to Medication Scale (Horne et al., 1997)	7,8	4
Satmed Q/ Satisfaction with Medication Questionnaire (Ruiz et al., 2008); French version (Delestras et al, 2013)	21,22	17
SEAMS/Self-efficacy for appropriate medication use scale (Risser et al., 2007)	13	13
SOC/Stage Of Change for Adherence Measure (Willey, 2000)	15	2
Tarquinio et al., 2000 French version	24	12
TOTAL		194

*References for the questionnaires are given in the supporting information S1 File.

**Table 2:** Classification of items found in literature review by WHO determinants of patient adherence to drug therapy compared with qualitative analysis.

Dimension	Theme	Patient	Health professionnal	Number of Items found in Literature	Number of Items in QUILAM
Disease	D-1 Disease/ Lack of symptoms	**X**	**X**	**8**	
	D-2 Comorbidity	**X**	**X**		
	D-3 Addictions (drugs, alcohol, tobacco)	**X**	**X**	**1**	
	D-4 Depressive disorders	**X**	**X**	**1**	
	D-5 Cognitive, visual and dexterity disorders	**X**	**X**	**3**	
Medication	M-1 Complexity of treatment	**X**	**X**	**4**	**1**
	M-2 Number of medication intakes	**X**	**X**	**3**	
	M-3 Number of drugs	**X**	**X**	**4**	**1**
	M-4 Objective adverse effect	**X**	**X**	**3**	
	M-5 Long term effect	**X**	**X**	**2**	
	M-6 Terms of drug administration/ methods of administration	**X**	**X**	**3**	
Demographic and socio-economic factors	DSEF-1- Material resources: income, job, poverty	**X**	**X**	**4**	
	DSEF-2- Age	**X**	**X**		
	DSEF-3- Social precarity, homelessness, immigrant status	**X**	**X**		
	DSEF-4- Ethnic identity				
	DSEF-5- Cultural identity	**X**	**X**		
Health care system	HCS – 1 Quality of the patient-care provider relationship	**X**	**X**	**4**	**3**
	HCS-2 Care organization	**X**	**X**	**4**	**1**
Patient and close relatives	P-1 Disease and medication knowledge	**X**	**X**	**5**	**1**
	P-2 Beliefs and attitudes towards the disease and medications	**X**	**X**	**27**	**1**
	P-3 Previous experiences (efficacy/ tolerance)	**X**	**X**	**24**	**2**
	P-4 Daily life	**X**	**X**	**10**	**1**
	P-5 External resources (social support, environment)	**X**	**X**	**7**	
	P-6 Internal resources	**X**	**X**	**2**	**1**
	P-7 Treatment self-management	**X**	**X**	**51**	**1**
	P-8 Behavior	**X**	**X**	**22**	**1**
	P-9 Healthy lifestyle (diet/ Physical Activity)	**X**	**X**	**2**	

#### Thematic analysis of the interviews.

All the dimensions of medication adherence proposed by the WHO and in the grid by Baudrant et al. [11] were found in the qualitative analysis of interviews of patients and healthcare providers (Table 2).

### 2. Reduction of number of items

#### Qualitative Reduction by an expert committee.

Forty-six convergent items were selected by at least 3 of the 5 experts (7 items selected by all 5 experts, 15 items by 4 experts and 24 items selected by 3 experts). The divergent items selected by only 2 experts were discussed and 16 of these were retained by consensus. Items were rejected due to lack of clinical relevance to chronic disease, as well as those that could be interpreted in several ways according to the experts, and items which were redundant. At the end of this stage 62 items remained (132 had been eliminated) in V1 of the questionnaire (see supporting information S1 File). As can be seen in Table 2, no items included in the final tool were related to the Disease or to the Demographic/socio-economic factors dimensions.

### Quantitative reduction

#### Study Population for quantitative reduction.

Among the 120 patients initially recruited for step three, 3 patients were excluded due to missing data, and one patient was excluded because it turned out that he did not meet the inclusion criteria. Therefore, 116 patients were asked to complete the 62-item version of the questionnaire (V1) and their responses analyzed. The characteristics of this hospital population is shown in Table 3.

**Table 3:** Characteristics of the patient populations used to test the questionnaires.

	Quantitative Reduction (V1)	Test-retest (V2)
**Number of patients**	116	**55**
**Age (year)**	64 (15)	**71 (12)**
**Sex, male**	72 (62.1%)	**38 (69.1%)**
**Level of education completed**		
Primary school	19 (16.4%)	4 (7.3%)
Middle school	45 (38.8%)	23 (41.8%)
High school	25 (21.6%)	11 (20.0%)
Undergraduate degree and higher	20 (17.2%)	13 (23.6%)
MD	7 (6.0%)	4 (7.3%)
**Marital status**		
Single	11 (9.5%)	4 (7.3%)
Married, civil partnership, cohabiting	74 (63.8%)	38 (69.1%)
Separated, divorced	19 (16.4%)	4 (7.3%)
Widowed	10 (8.6%)	8 (14.5%)
Missing data	2 (1.7%)	1 (1.8%)
**Situation at home**		
Living alone	41 (35.3%)	14 (25.5%)
Not living alone	74 (63.8%)	41 (72.7%)
Missing data	1 (0.9%)	1 (1.8%)
**Chronic disease**		
Type 2 Diabetes	41 (35.3%)	35 (63.6%)
COPD	36 (31.0%)	7 (12.7%)
Heart Failure	39 (33.6%)	18 (32.7%)
**Type of care**		
Full hospitalization	68 (58.6%)	0 (0.0%)
Day care	23 (19.8%)	0 (0.0%)
Regular consultations	25 (21.6%)	0 (0.0%)
Pharmacy refill	0 (0.0%)	55 (100%)
**Time since initiation of current treatment**		
0-2 months	0 (0.0%)	4 (7.3%)
2–6 months	19 (16.4%)	3 (5.5%)
6 months–1 year	3 (2.6%)	5 (9.1%)
1–5 years	25 (21.6%)	12 (21.8%)
6–10 years	22 (19.0%)	11 (20.0%)
>10 years	38 (32.8%)	19 (32.7%)
Missing data	9 (7.8%)	2 (3.6%)
**Medication management**		
Autonomous	100 (86.2%)	49 (89.1%)
Requiring assistance	15 (12.9%)	4 (7.3%)
Missing data	1 (0.9%)	2 (3.6%)
Number of medications	8(4)	8 (3)
Number of daily intakes	6(5)	3 (1)

For the question on the clarity and ease of answering the questionnaire, 68 patients (58%) answered “Yes, entirely” or “quite”, 23 “moderately” (20%), 13 “not very” (11%) and 6 “not at all” (5%). Six patients had missing data.

#### Analysis of responses for quantitative reduction.

An exploratory factorial analysis (EFA) reduced the QUILAM questionnaire to 31 items. After descriptive statistics, these 31 items with homogeneous distribution were selected and entered into the data matrix. The measure of sampling quality with the KMO index of the reduced scale was.661, which is acceptable. (see Table 4). The four dimensions retained for the final version of the QUILAM questionnaire were; *general beliefs*: items 8, 18, 10, 34 (α = .685), the *self-management of treatment* (unintentional non-compliance): items 56, 53, 49 (α = .653), the *specific beliefs concerning the treatment* (intentional non-compliance): items 46, 16, 43 (α = .667) and the *patient/health-care system relationship*: items 55, 54, 17, 39 (α = .598). The final fourteen item questionnaire (V2) is shown in S1 Table.

**Table 4:** Factor matrix from the exploratory factorial analysis during the reduction of V1 to the final version QUILAM V2, and associated dimensions.

	Factor				Dimension
1	2	3	4	Cronbach α
8.* Doctors prescribe too many treatments.	1.041				General beliefs α = .685
18. If doctors spent more time with patients, they would prescribe fewer treatments.	.600				
10. I sometimes worry about the long-term effects of my treatment.	.410		.304		
34. Natural remedies are safer than medical treatments	.340				
56. Sometimes I forget to refill my prescription(s).		.988			Self -management of the treatment (unintentional non-adhesion) α = .653
53. Sometimes I don’t have my medication with me at the time I’m supposed to take it.		.492			
49. I have difficulty managing all the medications I have to take.		.464			
46. Sometimes, for social reasons, I feel ill-at-ease taking my medication (e.g., when with friends).			.790		Specific beliefs about the treatment (intentional non-adhesion) α = .667
16. I am sometimes negligent in taking my medication.			.576		
43. I sometimes reduce or stop taking my medication without telling my doctor because I feel worse when I take it.			.539		
55. My doctor and I make decisions together				.765	Relationship between patient and the health care system α = .598
54. I understand my healthcare professional’s instructions about the medicines I take				.563	
17. My doctor (or other healthcare professional) explained to me how to properly treat my illness.				.478	
39. Overall, I am satisfied with this treatment				.388	
Shape Matrix with Convergence of Rotation in 5 iterations Extraction method: maximum likelihood/ Rotation method: Oblimin with Kaiser normalization. *The numbers refer to the question in V1 (see supporting information S1 File)					

### 3. Validation of the questionnaire and reliability over time

#### Test Re-test phase.

Fifty-six patients (community pharmacy clients) were included by 15 community pharmacies. Among the patients included, 35 had type 2 diabetics, 8 had COPD, and 18 had heart failure. Six patients had 2 pathologies (heart failure and type 2 diabetes) and 1 patient had 3 pathologies. One patient’s responses were excluded for lack of re-test at D15 (see Table 3).

The characteristics of the validation population (V2) are shown in Table 3. Out of the 55 patients, all considered the questionnaire acceptable, with only 2 items for which data were missing.

**Fig 3:**
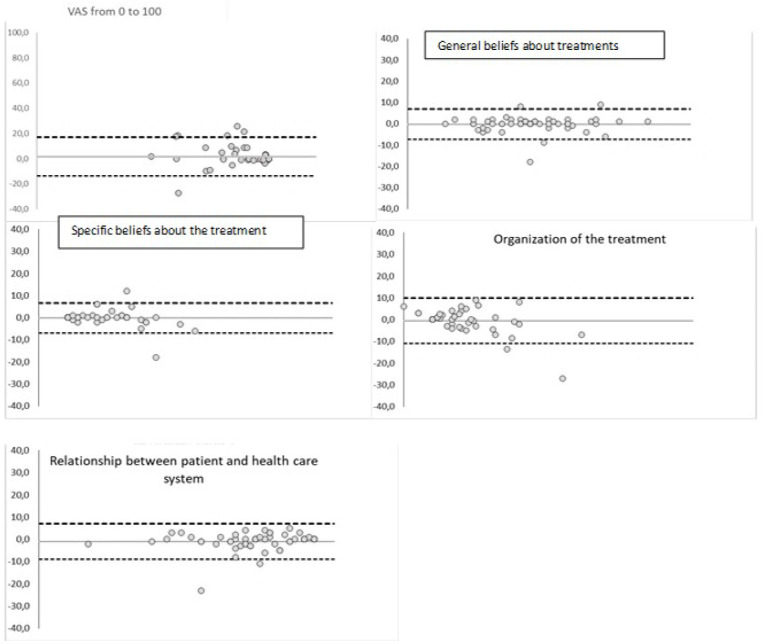
Bland Altman Test-retest.

Reliability results reported the Intra-class Correlation Coefficient (ICC) for each item in V2 used in the Test-retest stage (see Table 5). Fig 3 presents the graphic representations according to Bland-Altman for the VAS and for the different dimensions. Cronbach’s alpha calculated as an indication per dimension was 0.720 for the General Beliefs dimension, 0.706 for the Specific Beliefs dimension, 0.476 for the Self-management dimension and 0.697 for the patient/ healthcare professional relationship dimension.

**Table 5:** Intra-class correlation coefficient (ICC) per item in V2 test-retest.

	Test-Retest V2 – by Patients attending Community Pharmacies		
	N = 55	Intraclass correlation	95% confidence intervals
VAS from 0–100	55	.829	.723 −.897
Item 1	54	.762	.623 −.854
Item 2	55	.710	.549 −.820
Item 3	55	.675	.502 −.797
Item 4	55	.605	.409 −.748
Item 5	55	.721	.564−.827
Item 6	52	.785	.654−.871
Item 7	55	.603	.403−.748
Item 8	55	.528	.306−.695
Item 9	55	.642	.455−.775
Item 10	55	.688	.519−.806
Item 11	55	.769	.635−.858
Item 12	55	.756	.610−.851
Item 13	55	.743	.598−.842
Item 14	55	.665	.488−.790

ICC > 0.9: Excellent – 0.75 < ICC < 0.9 Good – 0.5 < ICC < 0.75 Moderate – ICC < 0.5 Poor.

According to Koo et Li,. 2016 [28].

## Discussion

To our knowledge, this is the first questionnaire available in English or French aimed at identifying the barriers and levers to medication adherence in patients with chronic diseases, in order to implement personalized interventions to improve patient outcomes.

The literature search associated with semi-structured interviews provided a large number and range of items for the qualitative analysis. In order to ensure that all dimensions of medication adherence defined by the WHO [7] were covered, a grid was used to classify the items [11]. After an initial pre-selection step to reduce the number of items, this was then further shortened by the panel of experts, before the questionnaire was tested on patients.

This gave an excessively large number of items (194) in the first version (V0). Given the high risk of measurement bias and the difficulties of interpretation of statistical analyses for a questionnaire of 194 items, reduction to 62 items was carried out by the investigators and then by a multidisciplinary committee of experts. The choice of items was made on the basis of clinical criteria by a panel of 8 experts from different fields. The anonymous pre-selection of items made it possible to avoid the weighting of opinions according to an opinion leader such that the eight members of the expert committee were able to debate until a consensus was reached using a Delphi method. However, to make the questionnaire acceptable to patients and as short as possible, the 62-item questionnaire (V1) was tried-out on patients, followed by an exploratory factor analysis of patient responses for the quantitative reduction of items. The five dimensions did not all emerge as factors in the factor matrix during this analysis. Compared to the determinants proposed by Sabaté [7] two dimensions were not retained “demographic and socio-economic factors” and “disease”, which cannot be acted on.

This reinforced our choice made concerning exploratory factor analysis. Indeed, the four dimensions retained for the final version of the QUILAM questionnaire (V2) were general beliefs about treatment, the self-management of treatment (unintentional non-compliance), specific beliefs about the treatment (intentional non-compliance) and the patient/health care system relationship. These dimensions are the ones we were interested in, targeting potential levers for medication adherence that can be “activated” through behavioral change [14].

The test-retest of V2 with community pharmacy patients found good acceptability of the questionnaire with little missing data. QUILAM can be repeated by the same person at two different times since the results had acceptable concordance [28]. The Cronbach’s alpha was low for the dimension on self-management of treatment, which may be explained by the fact that only 3 items explored this dimension. Although there is no consensus about the minimal value of Cronbach alphas, coefficients around 0.6 be could be considered as slightly low. This is close to other results obtained in similar situations, and could be explained by the limited number of questions per item.

A good correlation was found between the adapted Kalichman VAS and the total QUILAM score (.73). The QUILAM score is inverted in relation to Kalichman’s score: the lower the QUILAM score, the more adherent patients are.

As clinicians or coordinators of therapeutic patient education programs, this work has led, as intended, to the provision of a generic tool for use in routine clinical practice, whatever the chronic disease, that targets patients in difficulty with their drug treatment and helps to identify levers that could be mobilized for customized educational support. The QUILAM questionnaire could be used as a tool that captures the patient’s perception at a given time. It could be used before or during a regular consultation or a specific adherence visit as a mediator to initiate structured communication with the patient. Depending on the target of the contact with the patient, before using the QUILAM questionnaire the instructions he/she receives must be very clear, i.e., does it relate to a single condition (in a context of comorbidity) or a broad comorbid condition; does it target a specific drug or a complete prescription? This will help acceptation by the patient and limit the risk of a social desirability bias [29].

### Limitations

Due to its complexity the project took a long time. The literature might have evolved since we started, but the issue tackled here is still of interest: “*to identify modifiable factors relating to non-adherence which can then be the focus of adherence support interventions at the level of the individual*” [14]. People interviewed in 2013 and 2014 might give different responses today, as patient distrust toward medications has grown, and COVID-19 has altered their perceptions of illness and healthcare providers. However, the complex nature of medication non-adherence has probably not changed drastically in the past decade.

Concerning the Delphi process, the panel was composed of 8 experts. Although this is at the lower end of the number of experts usually suggested, we chose representatives from the broad range of different scientific disciplines concerned by this project (3 pharmacists, 1 general practitioner, 2 health psychologists, 1 educational psychology researcher and 1 social psychology researcher). At this stage we did not include patient representatives as the next stage of quantitative validation guaranteed consideration of the patients’ perspectives.

We chose to target three major highly prevalent and emblematic conditions, in order to address an extensive rather than just a representative spectrum of barriers. We endorsed a qualitative perspective, aiming to detect barriers at an individual level, using 20 scales measuring drug adherence validated in several pathologies, to help emblematic dimensions to emerge. Indeed, QUILAM is not designed for epidemiological purposes and doesn’t address representativity on a populational scale.

Quantitative reduction & Test-retest validation was not performed on the same profile of population (patients attending hospital consultations vs patients attending a community pharmacy). This was done to test the generalizability of the tool. For example, there were more patients with COPD during the quantitative reduction phase than the test-retest phase, which could be explained by the different times at which they were conducted, with a possible seasonal effect.

### Perspectives

At an individual level, the challenge to be met involves many determinants for each patient [30]. The need to identify patients’ difficulties is obvious, to help them find meaning in the management of their disease and treatment within their overall family, professional, cultural and social environment. It’s a prerequisite for the patient to identify and organize his or her own strategies for taking medication on a routine basis or in the face of day-to-day hazards. It is also a prerequisite for the healthcare team, with a view to co-construction with the patient. This is the context in which the QUILAM questionnaire is designed, to provide a routine tool to address the day-to-day problems faced by healthcare professionals in targeting and supporting patients most at risk.

The different phases of the medication adherence process: initiation, implementation and persistence [12,13] are targeted by this screening tool. The self-reported measurement format was chosen because, unlike electronic pillboxes, it is easy to implement on a large scale and inexpensive. This choice also makes it possible to adapt the measurement from a highly targeted assessment of a pathology or drug to an overall assessment of behavior.

Like the algorithm used in the MEMO drug monitoring and optimization project developed in the Netherlands [31] and the MEMS report used as the basis for a complex intervention model [32,33], the QUILAM questionnaire is designed to screen at-risk patients with difficulties but also patients who could potentially have difficulty taking their medication. It serves as a gateway to a targeted interview, enabling us to get to know the patient, his or her context and behavior.

We expect this tool to be able to describe the barriers and levers to medication adherence, so that we can then carry out a targeted intervention. Administering the questionnaire has no interventional impact as such, other than to provide patients with a moment of introspection, to take stock of their general and specific beliefs, how they organize themselves and their relationship with their various healthcare professionals. As the questions can be grouped according to 4 dimensions, different perspectives of intervention can be adapted to the obstacles and levers identified. As healthcare professionals (clinicians, pharmacists, psychologists etc.) we think that it vital to stop interpreting and treating the potential reasons for change on our own, and to turn to the patient himself/herself, asking what he/she is willing to change and how we can support them to find their own solution [34,35].

## Conclusion

The QUILAM questionnaire is simple and easily integrated into routine clinical practice. It helps structure adherence visits, targets patient’s own issues with medication and helps define a customized follow-up. QUILAM could potentially be integrated into electronic medical record systems to make it easily accessible during consultations.

## Supporting information

S1 File QUILAM qualitative interview guide, QUILAM Questionnaire V1, French version of questionnaire V2, and Literature search references. V1 is a translation of the original 62 item French version completed by French speaking patients before Quantitative reduction to V2 (14 items); the French version of V2 was the validated version; and references to questionnaires retained in the literature search (Table 1).
(DOCX)

S2 File Qualitative Reduction Raw Data.xls. QUILAM_Qualitative Reduction_Initial 194 items. Excel in Frenchx.
(XLSX)

S3 File Quantitative Reduction Raw Data.xlsx. QUILAM_Quantitative Reduction_Patient Characteristics and replies to V1. Excel in French.
(XLSX)

S4 File Test-retest Data.xlsx. QUILAM_test-retest D0-D15 Patient Characteristics and replies to V2. Excel in French.
(XLSX)

S1 Table QUILAM V2. Translation of original French questionnaire (Validated in French, not English).
(DOCX)
