# Supplementary material for: Development of a self-administered questionnaire to identify levers and barriers to adherence to medication regimens for chronic disease: The QUILAM project
Source: PLoS One. 2025 Oct 23;20(10):e0323542. doi: 10.1371/journal.pone.0323542 (PMC12548849; doi:10.1371/journal.pone.0323542)
Supplement: S1 File — V1 is a translation of the original 62 item French version completed by French speaking patients before Quantitative reduction to V2 (14 items); the French version of V2 was the validated version; and references to questionnaires retained in the literature search (Table 1). (DOCX) [file pone.0323542.s001.docx]

**Supporting information to:**

**Development of a self-administered Questionnaire to Identify Levers of Adherence to Medication regimens for chronic disease; the QUILAM project**

**Lehmann et al.**

**SUPPORTING INFORMATION**

**S1_Supporting Information (**this file)

- **QUILAM qualitative interview guide** (translated from French)
- **QUILAM Questionnaire V1.** Translation of the original 62 item French version completed by French speaking patients before Quantitative reduction to V2 (14 items)
- **QUILAM questionnaire V2** in French (this was the validated version)
- **Literature search references.** References to questionnaires retained in the literature search (see Table 1)

**S2, S3, S4 Raw Data (Separate Excel files in French)**

- **S2_ QUILAM_Qualitative Reduction_Initial 194 items.** [QUILAM_Réduction Qualitative_194 ITEMS A SELECTIONNER] (Excel in French)
- **S3_ QUILAM_Quantitative Reduction_Patient Characteristics and replies to V1.** [QUILAM_Réduction Quantitative Caractéristiques patients VI et réponses à V1] (Excel in French)
- **S4_ QUILAM_test-retest D0-D15 Patient Characteristics and replies to V2.** [QUILAM_test-retest J0-J15 Caractéristiques patients V2 et réponses à V2] (Excel in French)

**QUILAM QUALITATIVE INTERVIEW GUIDE** (translated from French)

- **Explain the purpose of the interview**

In order to improve the support, follow up and therapeutic care of patients, research work is currently being carried out by the pharmacy of Grenoble University Hospital and Lyon Civil Hospitals in collaboration with the INSERM Clinical Investigation Center and the pulmonology; cardiology, diabetology and cardiac rehabilitation departments.

The aim of this interview is to get to know you better and to talk about the difficulties you may encounter in relation to your illness and your medications.

If you agree, this consists of an interview of around thirty minutes. An audio recording of this interview will be made (if you agree) in order to facilitate its analysis.

- **Interview :**
- **Can you tell me about your heart failure / diabetes / COPD?**
- **Do you have any other health problems?**
- **At the moment, what concerns you most about your illness?**
- **What situations are causing you problems in your daily life?**
- **Outside of the hospital, who are the people in the medical world you trust to ask questions about your illness and your treatment?** (Your GP / Your specialist doctors / Your nurses / Your physiotherapists / Your dieticians / Your pharmacists / No one / Other(s) )
- **What are your other sources of information?** (Patient associations / Other patients / Books and magazines / Internet / None / Other(s))
- **What do you think of your treatments?**
  - What are their constraints?
  - their benefits?
- **Currently, what concerns you most about your treatment?**
- **How do you take your medication at home?**
- **What helps you to take your treatment at home?**
- **What are the difficulties you encounter on a daily basis with:**
  - managing the taking of the treatment every day
  - the times for taking the treatment …
  - the annoying effects …
  - the interactions with other medicines or foods
  - your supply of medicines
  - the management of your stocks of medicines …
  - fear of forgetting …
  - what to do in case of forgetting …
  - other difficulties …
- **Do you feel those around you understand and support you regarding your illnesss?**

**If so, how involved are those around you in managing your illness and/or your treatments**

- **What do you think of your relationship with:**
  - your hospital caregivers?
  - your community caregivers?
- **What is the impact of your illness and your treatment on your life??**

(Family life – Social life – Professional life – Leisure)

- **How do you take care of yourself on a daily basis in relation to your illness?**
- **What else would you like us to discuss together (in relation to your care)?**

**Thank you for your valuable help and the time you have given me. Would you like to add anything...**

***The healthcare professionals were asked the same questions, but about their patients (i.e., in the third person)***

**QUILAM V1: Translation of the original 62 item French version completed by French speaking patients before Quantitative reduction**

## **QUILAM QUESTIONNAIRE**

**Your Medications and You**

**It is often challenging to have to take medication regularly for a chronic illness. We would like to know about the difficulties you encounter in taking your treatment (medication) for your illness.**

**This questionnaire includes 4 pages. Please take the time to answer all of the following questions:**

**Part 1**

**On a scale of 0 to 100 (0 means you never take your medication, and 100 means you always take it on time and always the prescribed dose), place a cross on the black bar where you think you are.**

| **0** |  | **100** |
| --- | --- | --- |
|  | | |
| **I never take my medication** |  | **I always take all my medications, on time and at the prescribed dose** |

**Part 2**

**For each question, circle the answer that best suits you (from 1 = I strongly disagree, to 7 = I entirely agree)**

| **I strongly I entirely**  **disagree agree** | | |
| --- | --- | --- |
| 1. I only take my medication when I am sick | **0 1 2 3 4 5 6 7** | |
| 1. When I feel better, I sometimes stop taking my medication. | **0 1 2 3 4 5 6 7** | |
| 1. Last month I felt sad, depressed, or down | **0 1 2 3 4 5 6 7** | |
| 1. I have already not always taken my medication because some days my memory fails me. | **0 1 2 3 4 5 6 7** | |
| 1. Generally, my treatment is easy to take/use in its current form (taste, size, etc.). | **0 1 2 3 4 5 6 7** | |
| 1. It is inconvenient to take medication more than once a day. | **0 1 2 3 4 5 6 7** | |
| 1. I have too many medications to take each day | **0 1 2 3 4 5 6 7** | |
| 1. Doctors prescribe too many treatments. | **0 1 2 3 4 5 6 7** | |
| 1. I am confident in my ability to take my medications as prescribed when I have side effects. | **0 1 2 3 4 5 6 7** | |
| 1. I sometimes worry about the long-term effects of my treatment. | **0 1 2 3 4 5 6 7** | |
| 1. It is difficult for me to swallow the medications I have to take | **0 1 2 3 4 5 6 7** | |
| 1. I have difficulty “handling” my treatments (taking out the medication, opening the box, opening the blister, handling my inhaler, giving myself injections, etc.). | **0 1 2 3 4 5 6 7** | |
| 1. I have already missed a dose, not refilled my medication, or taken less medication because of its cost. | **0 1 2 3 4 5 6 7** | |
| 1. I sometimes skip a medication to see if I still need it. | **0 1 2 3 4 5 6 7** | |
| 1. The treatment I am being offered is not suited to my lifestyle. | **0 1 2 3 4 5 6 7** | |
| 1. I am sometimes negligent in taking my medication. | **0 1 2 3 4 5 6 7** | |
| 1. My doctor (or other health professional) explained to me how to properly treat my illness. | **0 1 2 3 4 5 6 7** | |
| 1. If doctors spent more time with patients, they would prescribe fewer treatments. | **0 1 2 3 4 5 6 7** | |
| 1. I am confident in my ability to take my medications as prescribed when the doctor changes my treatment. | **0 1 2 3 4 5 6 7** | |
| 1. I am confident in my ability to take my medications as prescribed even though some pills look different from the ones I usually take. | **0 1 2 3 4 5 6 7** | |
| 1. I intend to continue this treatment | **0 1 2 3 4 5 6 7** | |
| 1. I am confident in my ability to take my medications as prescribed when I am unsure of what time of day to take my medications. | **0 1 2 3 4 5 6 7** | |
| 1. I am not sure how to take this medicine. | **0 1 2 3 4 5 6 7** | |
| 1. I am convinced of the importance of my medication. | **0 1 2 3 4 5 6 7** | |
| 1. I'm afraid my medication will do more harm than good. | **0 1 2 3 4 5 6 7** | |
| 1. I have already skipped or stopped taking a medication because I found it wasn't working | **0 1 2 3 4 5 6 7** | |
| 1. My health today depends on my treatment. | **0 1 2 3 4 5 6 7** | |
| 1. My future health depends on my treatment | **0 1 2 3 4 5 6 7** | |
| 1. Having to take medication worries me. | **0 1 2 3 4 5 6 7** | |
| 1. Without my treatment, I would be very ill. | **0 1 2 3 4 5 6 7** | |
| 1. I sometimes worry about becoming too dependent on my treatment. | **0 1 2 3 4 5 6 7** | |
| 1. Staying on treatment will prevent me from getting sick. | **0 1 2 3 4 5 6 7** | |
| 1. People taking medication should stop their treatment from time to time. | **0 1 2 3 4 5 6 7** | |
| 1. Natural remedies are safer than medical treatments. | **0 1 2 3 4 5 6 7** | |
| 1. I have confidence that each of my medications will help me. | **0 1 2 3 4 5 6 7** | |
| 1. It is not natural for my body and mind to be controlled by medication. | **0 1 2 3 4 5 6 7** | |
| 1. I do not consider taking this medication to be a priority in my daily routine. | **0 1 2 3 4 5 6 7** | |
| 1. Medicines make me tired and sleepy | **0 1 2 3 4 5 6 7** | |
| 1. Overall, I am satisfied with the treatment. | **0 1 2 3 4 5 6 7** | |
| 1. The treatment I am currently taking relieves my symptoms. | **0 1 2 3 4 5 6 7** | |
| 1. I feel better now than before I started treatment. | **0 1 2 3 4 5 6 7** | |
| 1. I feel well with this treatment | **0 1 2 3 4 5 6 7** | |
| 1. I sometimes reduce or stop taking my medication without telling my doctor because I feel worse when I take it. | **0 1 2 3 4 5 6 7** | |
| 1. The side effects of the treatment have an impact on my daily tasks. | **0 1 2 3 4 5 6 7** | |
| 1. I’m afraid that my medications will affect my sexual health. | **0 1 2 3 4 5 6 7** | |
| 1. Sometimes, for social reasons, I feel ill at ease taking my medication (e.g. when I’m with friends). | **0 1 2 3 4 5 6 7** | |
| 1. I am confident in my ability to take my medications as prescribed when my usual routine is disrupted. | **0 1 2 3 4 5 6 7** | |
| 1. I had difficulty doing what the doctor advised me to do. | **0 1 2 3 4 5 6 7** | |
| 1. I have difficulty managing all the medications I have to take. | **0 1 2 3 4 5 6 7** | |
| 1. I have someone I can call with questions about medications. | **0 1 2 3 4 5 6 7** | |
| 1. I am confident in my ability to take my medications as prescribed even if no one reminds me to take them. | **0 1 2 3 4 5 6 7** | |
| **Never** | | **Always** |
| 1. I sometimes take my medication late compared to the usual time. | **0 1 2 3 4 5 6 7** | |
| 1. Sometimes I don't have my medications with me at the time I'm supposed to take them. | **0 1 2 3 4 5 6 7** | |
| 1. I understand my healthcare professional’s instructions about the medicines I take | **0 1 2 3 4 5 6 7** | |
| 1. My doctor and I make decisions together. | **0 1 2 3 4 5 6 7** | |
| 1. Sometimes I forget to refill my prescription(s). | **0 1 2 3 4 5 6 7** | |
| 1. I agree to biological, clinical or other examinations. | **0 1 2 3 4 5 6 7** | |
| 1. I sometimes skip a dose of medication or adjust it according to my own needs. | **0 1 2 3 4 5 6 7** | |
| 1. I sometimes forget to take my medication. | **0 1 2 3 4 5 6 7** | |
| 1. I keep medical appointments. | **0 1 2 3 4 5 6 7** | |
| 1. I have a healthy and balanced diet. | **0 1 2 3 4 5 6 7** | |
| 1. I have a healthy life | **0 1 2 3 4 5 6 7** | |

**In your opinion, is this questionnaire clear and easy to complete?**

| **🞏 No, not at all** | **🞏 Not very** | **🞏 Moderately** | **🞏 Quite** | **🞏 Yes, entirely** |
| --- | --- | --- | --- | --- |

**Thank you for your participation**

**QUILAM questionnaire (V2) in French (validated version)**

## **QUESTIONNAIRE QUILAM**

**Vos médicaments et vous**

**Il est souvent difficile de devoir prendre des médicaments régulièrement pour une maladie chronique. Nous cherchons à connaître les difficultés que vous rencontrez dans la prise de votre traitement (de vos médicaments) pour votre maladie.**

**Ce questionnaire comprend 2 pages. Merci de prendre le temps de répondre à toutes les questions suivantes**

**Première partie**

**Sur une échelle de 0 à 100 (0 signifie que vous ne prenez jamais vos médicaments, et 100 que vous les prenez toujours, à l’heure et 0 la dose prescrites), placez une croix sur la barre noire là où vous estimez vous situer.**

| **0** |  | **100** |
| --- | --- | --- |
|  | | |
| **Vous ne prenez jamais  vos médicaments** |  | **Vous prenez toujours tous vos médicaments,  à l’heure et à la dose prescrite** |

**Deuxième partie**

**Pour chaque question, entourez la réponse qui vous correspond le mieux de 1 à 7 (1 = pas du tout d’accord, 7 = Tout à fait d’accord)**

Pas du tout

d’accord

Tout à fait

d’accord

| 1. 1 | 1. Les médecins utilisent trop de médicaments. | | | | | **1 2 3 4 5 6 7** | |
| --- | --- | --- | --- | --- | --- | --- | --- |
| 1. 2 | 1. Je m'inquiète parfois à propos des effets à long terme de mon traitement. | | | | | **1 2 3 4 5 6 7** | |
| 1. 3 | 1. Je suis parfois négligeant(e) dans la prise de mes médicaments | | | | | **1 2 3 4 5 6 7** | |
| 1. 4 | 1. Mon médecin (ou autre professionnel de santé) m’a expliqué comment traiter correctement ma maladie. | | | | | **1 2 3 4 5 6 7** | |
| 1. 5 | 1. Si les médecins passaient plus de temps avec les patients, ils prescriraient moins de médicaments. | | | | | **1 2 3 4 5 6 7** | |
| 1. 6 | 1. Les remèdes naturels sont plus sûrs que les traitements médicaux. | | | | | **1 2 3 4 5 6 7** | |
| 1. 7 | 1. Je suis globalement satisfait(e) de ce traitement. | | | | | **1 2 3 4 5 6 7** | |
| 1. 8 | 1. Il m’arrive de diminuer ou d’arrêter de prendre mes médicaments sans le dire à mon médecin, parce que je me sens plus mal lorsque je les prends. | | | | | **1 2 3 4 5 6 7** | |
| 1. 9 | 1. Il m’arrive de ne pas être à l’aise pour prendre mon médicament pour des raisons sociales (ex : j’étais avec des amis). | | | | | **1 2 3 4 5 6 7** | |
| 10 | J’ai des difficultés à gérer tous les médicaments que je dois prendre. | | | | | **1 2 3 4 5 6 7** | |
| **(1 =** Jamais**, 7 =** Systématiquement**)** | |  |  |  | Jamais  Jamais | | Systématiquement |
| 11 | Il m’arrive de ne pas avoir mes médicaments avec moi au moment où je devais les prendre. | | | | | **1 2 3 4 5 6 7** | |
| 12 | Je comprends les instructions données par les professionnels de santé sur la prise des médicaments. | | | | | **1 2 3 4 5 6 7** | |
| 13 | Nous prenons les décisions ensemble avec mon médecin. | | | | | **1 2 3 4 5 6 7** | |
| 14 | Il m'arrive d'oublier de faire renouveler mes prescriptions. | | | | | **1 2 3 4 5 6 7** | |

**Nous vous remercions de votre collaboration**

**REFERENCES TO QUESTIONNAIRES RETAINED IN THE LITERATURE SEARCH (Table 1)**

| 1. **Morisky DE, Green LW, Levine DM. Concurrent and predictive validity of a self-reported measure of medication adherence. Med Care. Jan 1986;24(1):67‑74.** |
| --- |
| 1. **Thompson K, Kulkarni J, Sergejew AA. Reliability and validity of a new Medication Adherence Rating Scale (MARS) for the psychoses. Schizophr Res. 5 May 2000;42(3):241‑247.** |
| 1. **Korb-Savoldelli V, Sabatier B, Gillaizeau F, Guillemain R, Prognon P, Bégué D, et al. Non-adherence with drug treatment after heart or lung transplantation in adults: a systematic review. Patient Educ Couns. 2010;81(2):148‑154.** |
| 1. **Brooks CM, Richards JM, Kohler CL, Soong SJ, Martin B, Windsor RA, et al. Assessing adherence to asthma medication and inhaler regimens: a psychometric analysis of adult self-report scales. Med Care. Mar 1994;32(3):298‑307.** |
| 1. **Kripalani S, Risser J, Gatti ME, Jacobson TA. Development and evaluation of the Adherence to Refills and Medications Scale (ARMS) among low-literacy patients with chronic disease. Value Health J Int Soc Pharmacoeconomics Outcomes Res. Feb 2009;12(1):118‑123.** |
| 1. **Choo PW, Rand CS, Inui TS, Lee ML, Cain E, Cordeiro-Breault M, et al. Validation of patient reports, automated pharmacy records, and pill counts with electronic monitoring of adherence to antihypertensive therapy. Med Care. Sept 1999;37:846‑57.** |
| 1. **Horne R. Representations of medication and treatment: Advances in Theory and Measurement. Perceptions of Health and Illness: Current Research and Applications. Ed. Petrie, K.J. and Weinman, J. London: Harwood Academic; 1997. p. 155‑187.** |
| 1. **Horne R, Weinman J, Hankins M. The beliefs about medicines questionnaire: The development and evaluation of a new method for assessing the cognitive representation of medication. Psychol Health. 1999;14(1):1‑24.** |
| 1. **Hahn SR, Park J, Skinner EP, Yu-Isenberg KS, Weaver MB, Crawford B, et al. Development of the ASK-20 adherence barrier survey. Curr Med Res Opin. July 2008;24(7):2127‑2138.** |
| 1. **Matza LS, Park J, Coyne KS, Skinner EP, Malley KG, Wolever RQ. Derivation and validation of the ASK-12 adherence barrier survey. Ann Pharmacother. Oct 2009;43(10):1621‑1630.** |
| 1. **Unni EJ, Olson JL, Farris KB. Revision and validation of Medication Adherence Reasons Scale (MAR-Scale). Curr Med Res Opin. 30 Oct 2013;** |
| 1. **Girerd X, Hanon O, Anagnostopoulos K, Ciupek C, Mourad JJ, Consoli S. Assessment of antihypertensive compliance using a self-administered questionnaire: development and use in a hypertension clinic. Presse Médicale. 2001;30(21):1044‑1048.** |
| 1. **Risser J, Jacobson TA, Kripalani S. Development and psychometric evaluation of the Self-efficacy for Appropriate Medication Use Scale (SEAMS) in low-literacy patients with chronic disease. J Nurs Meas. 2007;15(3):203‑219.** |
| 1. **Walsh JC, Mandalia S, Gazzard BG. Responses to a 1 month self-report on adherence to antiretroviral therapy are consistent with electronic data and virological treatment outcome. AIDS Lond Engl. 25 Jan 2002;16(2):269‑277.** |
| 1. **Willey C, Redding C, Stafford J, Garfield F, Geletko S, Flanigan T, et al. Stages of change for adherence with medication regimens for chronic disease: development and validation of a measure. Clin Ther. July 2000;22(7):858‑871.** |
| 1. **Schroeder K, Fahey T, Hay AD, Montgomery A, Peters TJ. Adherence to antihypertensive medication assessed by self-report was associated with electronic monitoring compliance. J Clin Epidemiol. June 2006;59(6):650‑651.** |
| 1. **Fall E, Gauchet A, Cyril Tarquinio, Horne R. Validation of the French version of the Beliefs about Medicines Questionnaire (BMQ) among diabetes and HIV patients. European Review of Applied Psychology. 2014;64**[**:**](https://www.sciencedirect.com/journal/european-review-of-applied-psychology/vol/64/issue/6)**335-343.** |
| 1. **Gauchet A, Tarquinio C, Fischer G. Psychosocial predictors of medication adherence among persons living with HIV. Int J Behav Med. 2007;14(3):141‑150.** |
| 1. **McHorney CA, Victor Spain C, Alexander CM, Simmons J. Validity of the adherence estimator in the prediction of 9-month persistence with medications prescribed for chronic diseases: a prospective analysis of data from pharmacy claims. Clin Ther. Nov 2009;31(11):2584‑2607.** |
| 1. **Hogan TP, Awad AG, Eastwood R. A self-report scale predictive of drug compliance in schizophrenics: reliability and discriminative validity. Psychol Med. Feb 1983;13(1):177‑183.** |
| 1. **Ruiz MA, Pardo A, Rejas J, Soto J, Villasante F, Aranguren JL. Development and validation of the « Treatment Satisfaction with Medicines Questionnaire » (SATMED-Q). Value Health J Int Soc Pharmacoeconomics Outcomes Res. 2008;11(5):913‑926.** |
| 1. **Delestras S, Roustit M, Bedouch P, Minoves M, Dobremez V, Mazet R, et al. Comparison between two generic questionnaires to assess satisfaction with medication in chronic diseases. PLoS One. 2013;8(2):e56247.** |
| 1. **Kravitz RL, Hays RD, Sherbourne CD, DiMatteo MR, Rogers WH, Ordway L, et al. Recall of recommendations and adherence to advice among patients with chronic medical conditions. Arch Intern Med. 23 Aug 1993;153(16):1869‑1878.** |
| 1. **Tarquinio, C., Fischer, G.N., & Grégoire, A. Compliance in HIV-positive patients: validation of a French scale and measurement of psychosocial variables. Revue international de psychologie sociale 2000, 13(2), 61-91.** |
