# Supplementary material for: Development of a self-administered questionnaire to identify levers and barriers to adherence to medication regimens for chronic disease: The QUILAM project
Source: PLoS One. 2025 Oct 23;20(10):e0323542. doi: 10.1371/journal.pone.0323542 (PMC12548849; doi:10.1371/journal.pone.0323542)
Supplement: S1 Table — Translation of original French questionnaire (Validated in French, not English). (DOCX) [file pone.0323542.s005.docx]

**Table 5. QUILAM V2**

***Translation of original French questionnaire (Validated in French, not English)***

## **QUILAM QUESTIONNAIRE**

**Your Medications and You**

**It is often challenging to have to take medication regularly for a chronic illness. We would like to know about the difficulties you encounter in taking your treatment (medication) for your illness.**

**This questionnaire includes 2 pages. Please take the time to answer all of the following questions:**

**Part 1**

**On a scale of 0 to 100 (0 means you never take your medication, and 100 means you always take it on time and always the prescribed dose), place a cross on the black bar where you think you are.**

| **0** |  | **100** |
| --- | --- | --- |
|  | | |
| **I never take my medication** |  | **I always take all my medications, on time and at the prescribed dose** |

**Part 2**

**For each question, circle the answer that best suits you (1 = I strongly disagree, 7 = I entirely agree)**

Entirely

agree

Strongly

disagree

| 1. 1 | 1. Doctors prescribe too many medications | **1 2 3 4 5 6 7** |
| --- | --- | --- |
| 1. 2 | 1. I sometimes worry about the long-term effects of my treatment | **1 2 3 4 5 6 7** |
| 1. 3 | 1. I am sometimes negligent in taking my medication | **1 2 3 4 5 6 7** |
| 1. 4 | 1. My doctor (or other health professional) explained to me how to properly treat my illness | **1 2 3 4 5 6 7** |
| 1. 5 | 1. If doctors spent more time with patients, they would prescribe fewer medications | **1 2 3 4 5 6 7** |
| 1. 6 | 1. Natural remedies are safer than medical treatments | **1 2 3 4 5 6 7** |
| 1. 7 | 1. Overall, I am satisfied with my current treatment | **1 2 3 4 5 6 7** |
| 1. 8 | 1. I sometimes reduce or stop taking my medication without telling my doctor because I feel worse when I take it | **1 2 3 4 5 6 7** |
| 1. 9 | 1. Sometimes, for social reasons, I feel ill-at-ease taking my medication (e.g. when with friends). | **1 2 3 4 5 6 7** |
| 10 | I have difficulty managing all the medications I have to take | **1 2 3 4 5 6 7** |

Always

Never

**(1 = never, 7 = always)**

| 11 | Sometimes I don't have my medications with me at the time I'm supposed to take them. | **1 2 3 4 5 6 7** |
| --- | --- | --- |
| 12 | I understand the instructions given by healthcare professionals on taking medications | **1 2 3 4 5 6 7** |
| 13 | My doctor and I make decisions together | **1 2 3 4 5 6 7** |
| 14 | I sometimes forget to refill my prescription(s) | **1 2 3 4 5 6 7** |

**Thank you for your participation**
